# A Network Analysis of the Impact of the Coronavirus Pandemic on the US Economy: A Comparison of the Return and the Momentum Picture

**DOI:** 10.3390/e27020148

**Published:** 2025-02-01

**Authors:** Janusz Miśkiewicz

**Affiliations:** 1 Institute of Theoretical Physics, University of Wrocław, pl. M. Borna 6, 50-204 Wrocław, Poland; janusz.miskiewicz@uwr.edu.pl; 2 Physics and Biophysics Department, Wrocław University of Environmental and Life Sciences, ul. Norwida 25, 50-375 Wrocław, Poland

**Keywords:** time series analysis, network analysis, time series distances, clustering analysis, cross-correlations

## Abstract

This study examines a cross-correlation analysis of companies included in the S&P 500 Index at three different intervals: before, during, and after the pandemic’s onset. The aim is to evaluate how the pandemic and related governmental actions have affected market structures and economic conditions. This paper introduces the notion of momentum time series, integrating return and volume data. We show that these momentum time series provide unique insights that differ from return time series, suggesting their potential utility in economic analysis. Our analysis employs the Manhattan and Mantegna distances to construct a threshold-based network, which we subsequently scrutinize. Lastly, we evaluate how the pandemic has influenced these outcomes.

## 1. Introduction

The worldwide spread of the new coronavirus (SARS-CoV-2) has significantly affected stock markets. However, this was not a conventional financial market crisis. A unique aspect of the crisis triggered by the emergence of SARS-CoV-2 was its level of predictability. The virus’s transmission, the rising number of cases, and actions taken by other nations allowed for some anticipation of what was to come. On the other hand, a similar scenario, where the crisis symptoms are properly recognized in advance, cannot be excluded in the future. Thus, understanding the changes brought by this exceptional event is essential. Furthermore, it is important to note that the market’s response was influenced by government-imposed restrictions and actions, whereas in a standard crisis, governments usually react to market conditions. This situation leads to another question about whether the market returns to its previous state after the special measures are abandoned.

This paper examines the correlation structure among major US companies, focusing on the S&P 500 Index components over three periods: before, during, and after the COVID-19 pandemic. The conventional stock market analysis is based on the share return due to their scalability, which makes it possible to estimate potential gains or losses for all investor types. However, this method does not accurately capture the market’s true condition. During crises, media reports frequently highlight the considerable losses faced by individuals, yet these asset changes are somewhat abstract as they simply involve multiplying the return by the quantity of shares owned, without any actual asset transactions by the investor. In reality, the true state of the market is better reflected by volume. This paper thus explores the interplay between returns and market momentum. The key aspect of momentum is that it incorporates both the price change and the corresponding volume response.

The stock market, like the economy in general, is a complex system featuring numerous interacting entities. It is not sufficient to investigate their mutual relationship. The network features must also be considered. However, the typical correlation analysis results in the generation of a correlation matrix (see, for example, [1,2,3,4,5]) or a distance matrix [6,7,8,9] representing a completely connected network, which is a rather basic result from the point of view of the network theory. Consequently, the correlation analysis is followed by the construction of a network. The minimum spanning tree (MST) is the most frequently used structure in current research [10,11]. The MST has been used in many studies (e.g., [12,13,14,15,16,17]). The main advantage of the MST is that it allows for properly categorizing companies into industry sectors [12,18,19,20,21]. The MST is also a valuable tool in the context of portfolio analysis, as it allows for the identification of a core structure within the market. However, the application of the MST inherently imposes restrictions on network configuration. The evident consequences of the tree structure are the absence of loops, cliques, etc. Accordingly, this paper employs an alternative network construction strategy. Instead of minimizing the sum of distances, the companies are categorized based on their correlation measure value: strongly, typically, and weakly correlated firms. The advantage of a threshold-based network is that the structure is not externally imposed, allowing for the observation of structural shifts.

**Figure 1:**
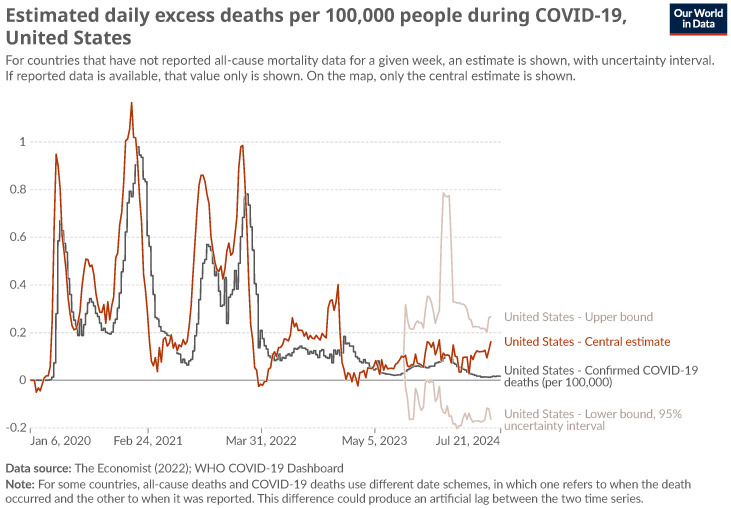
Time evolution of COVID-19 pandemic in USA. Source: https://ourworldindata.org/ (accessed on 9 August 2024).

Although the COVID-19 pandemic is recent in history, it seems reasonable to recall the crucial facts. The global pandemic caused by SARS-CoV-2, which is responsible for the illness known as coronavirus disease 2019 (COVID-19), originated in Wuhan, China, in December 2019. The virus rapidly spread worldwide, reaching the USA in January 2020. A comprehensive account of the epidemic’s progression in the USA can be found in [22]. The evolution of daily cases is presented in Figure 1. The analysis is based on data from the following three distinct time frames, from 1 March to the 1 June, across different years:

**Pre-COVID:** before COVID-19 pandemic in 2018;
**COVID:** during COVID-19 pandemic in 2020;
**Post-COVID:** after COVID-19 pandemic in 2022.

Each time frame consists of 63 data points, chosen from similar times of the year to reduce seasonal fluctuations. The progression of the pandemic is illustrated by the daily graph of new cases in Figure 1. It is notable that the United States experienced an increase in novel coronavirus cases in January 2020, followed by a gradual decline in March 2022. The initial period, designated as “pre-COVID”, is notable for its absence of the effects of the pandemic, as well as a lack of global crises, rendering it a suitable reference point. During the “COVID” period, the pandemic had already developed in the USA. The number of new daily cases was on the rise, and restrictions were being introduced in response. Consequently, companies must adapt to the evolving situation. It is crucial to recognize that this was not the inaugural instance of the advent of the SARS-CoV-2 pandemic. Instead, companies had been operating within novel circumstances for a period of 2 months, and thus, one might anticipate a certain degree of acclimation to the altered environment. The final period, termed “post-COVID”, encompasses the time when the number of new cases declined significantly and the original pandemic restrictions were relaxed. It seems reasonable to posit that companies have adapted to the new situation, and thus it is appropriate to analyze the network structure at the final stage of the pandemic.

**Figure 2:**
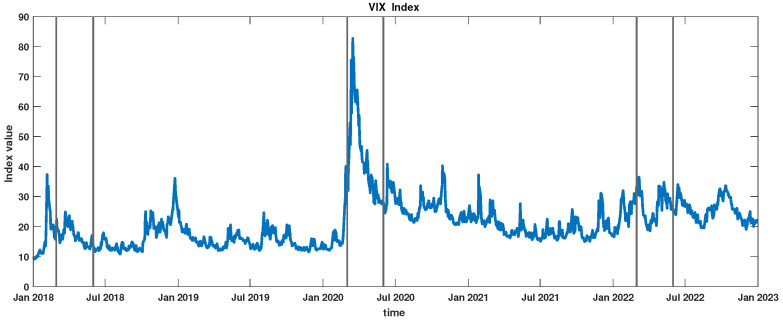
VIX Index in the interval 2018–2023. The considered periods, i.e., pre-COVID, COVID, and post-COVID, are marked by vertical lines.

The described sequence of events was reflected in investors’ sentiments. This fact is well illustrated by the evolution of the VIX Index. This index serves as a barometer of market uncertainty, offering insights into the anticipated 30-day future volatility. It is derived from options of the S&P 500 Index [23]. Figure 2 depicts the fluctuations of the VIX Index from 2018 to 2023. Historical data for the VIX Index can be found on the following Cboe website: https://www.cboe.com/tradable_products/vix/vix_historical_data/ (accessed on 30 December 2024). The intervals considered in this study are marked by vertical lines. Notably, the period labeled as COVID era stands out. The pronounced rise in volatility expectations on the New York Stock Exchange during this time underscores the heightened anxiety and uncertainty induced by the onset of the COVID pandemic, marking a phase when companies vigorously adapted to new conditions.

## 2. Data

The data were obtained from the Yahoo web page [24], involving daily values of S&P 500 Index components from the New York Stock Exchange. First, the data were verified against missing values, and companies with such missing values were removed from further analysis. After data verification, 493 entities were retained for further analysis. A comprehensive list of these companies can be found in Appendix A. The standard stock market abbreviations are used. The closing value time series are converted into the return time series in the following Equation (1):

(1) $$r\left(t\right)=\frac{p(t)-p(t-1)}{p(t-1)}$$

where p(t) denotes the share value at time *t*, while r(t) signifies the share return.

The return value (Equation (1)) reveals details about price variability but fails to account for how the market reacts to such changes. Accordingly, the concept of momentum is introduced in Equation (2). This idea stems from classical mechanics, where an object’s dynamics are characterized by the product of its mass and velocity, termed momentum. In a financial context, velocity can be related to the return described in Equation (1), which quantifies price variations over time. The mass is effectively denoted by the volume of shares traded in the market. The main advantage of this newly established variable is that it integrates both price changes and trading volumes of financial markets. From a macroeconomic viewpoint, it is crucial to address price shifts, whereas the market response, indicated by volume, holds significant importance.

(2) m(t)=r(t)·V(t)

where V(t) is the volume.

The main feature of the proposed measures (Equation (2)) is the inclusion of transaction volume, which, while not frequently analyzed, is vital for market assessment. These metrics (Equation (2)) can be aligned with theories of price formation. Traditional price theories view the price as the balance of an asset’s supply and demand [25,26]. Naturally, transaction volume is influenced by the price [27]. Consequently, volume serves as an indicator of how the market price is accepted by traders. A high exchange volume suggests that the price is deemed beneficial for both the seller and the buyer. Contemporary stock market price theories [28,29,30,31], utilizing stochastic processes and game theory [32,33], emphasize price evolution, often treating transaction volume as a secondary concern, though it is discussed in the literature. Notably, price theories based on stochastic processes typically overlook transaction volume, concentrating instead on pricing trends, akin to an infinite capacity market. However, empirical market data [34,35,36,37] reveal that price and volume are interdependent variables. Indeed, stock markets are structured to optimize transaction volume. Brokers gather offers with ask and bid prices and execute them at rates that maximize transaction volume. Naturally, offers can vary fixed price, current price, opening price, etc. Nonetheless, the core impact of stock exchange operations is transaction volume, with the magnitude of executed deals reflecting stock market consensus.

**Figure 3:**
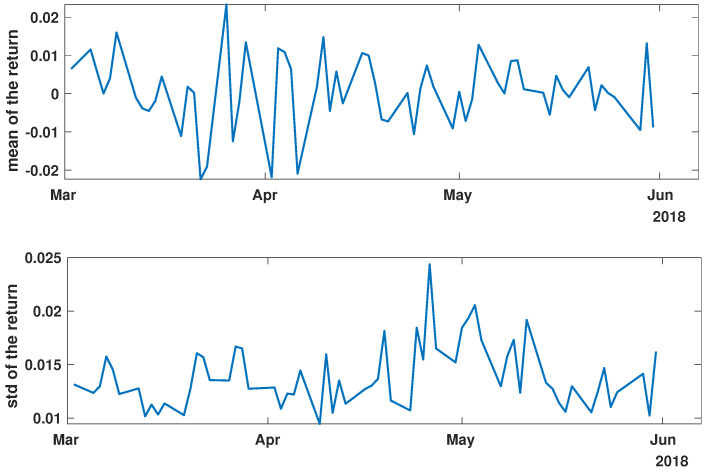
Time evolution of the mean value and standard deviation of the return of the analyzed companies’ stock market shares’ value before the COVID-19 crisis (*pre-COVID*).

**Figure 4:**
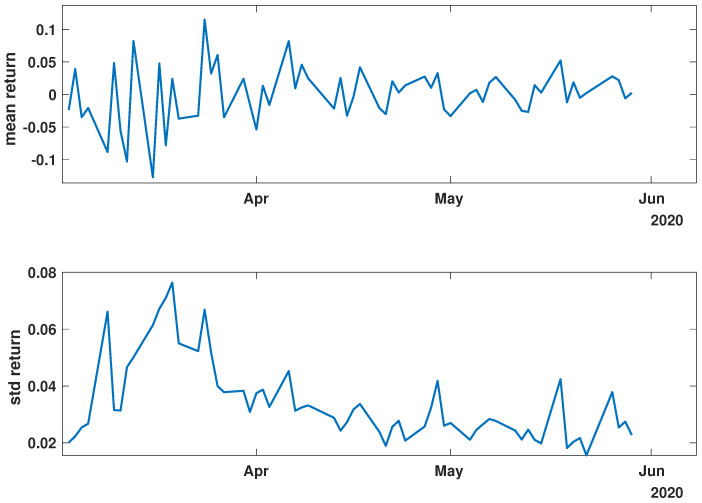
Time evolution of the mean value and standard deviation of the return of the analyzed companies’ stock market shares’ value during the COVID-19 crisis (*COVID*).

**Figure 5:**
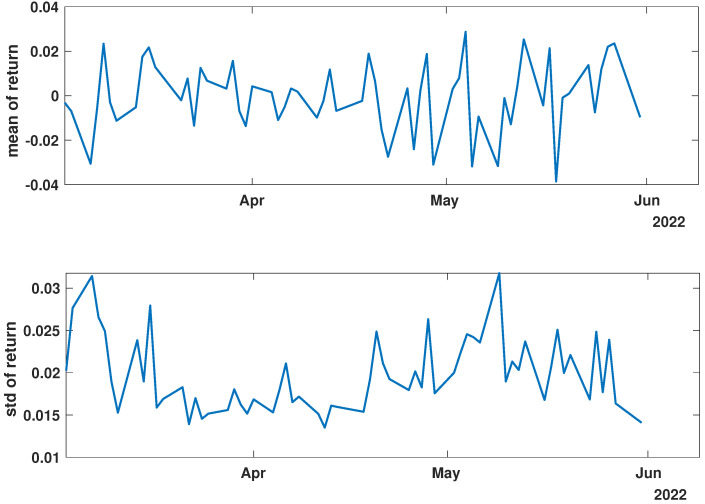
Time evolution of the mean value and standard deviation of the return of the analyzed companies’ stock market shares’ value after the COVID-19 crisis (*post-COVID*).

**Figure 6:**
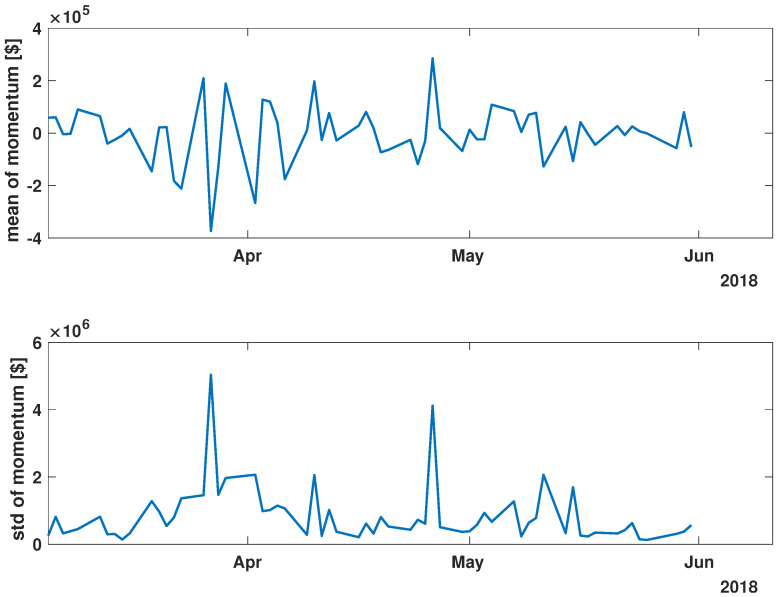
Time evolution of the mean value and standard deviation of the momentum of the analyzed companies’ stock market shares before the COVID-19 crisis (*pre-COVID*).

**Figure 7:**
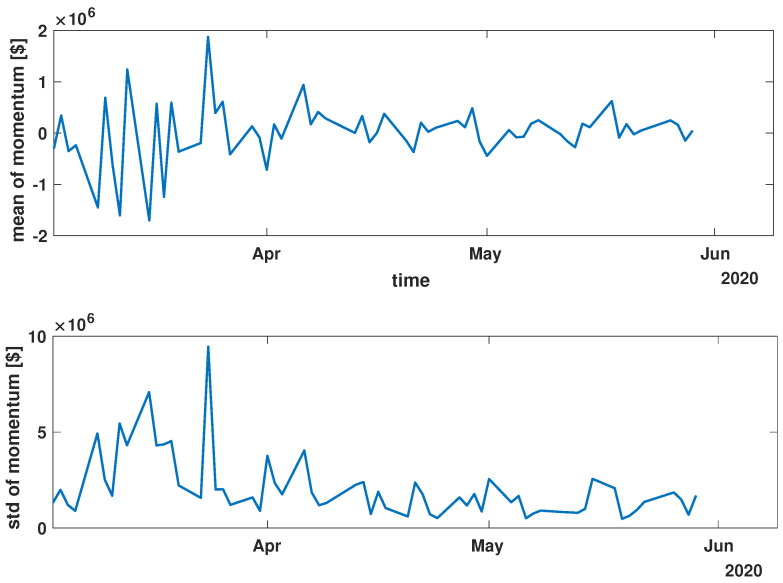
Time evolution of the mean value and standard deviation of the momentum of the analyzed companies’ stocks market shares during the COVID-19 crisis (*COVID*).

**Figure 8:**
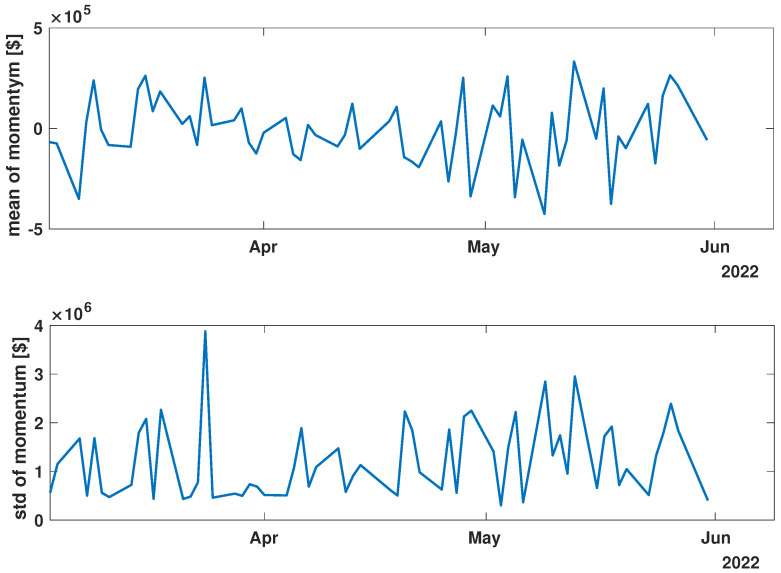
Time evolution of the mean value and standard deviation of the momentum of the analyzed companies’ stock market shares after the COVID-19 crisis (*post-COVID*).

In order to present the general characteristics of the studied time series, the mean and standard deviation of the returns and momentum of the group of time series were calculated. The evolutions of the mean and standard deviation at selected time intervals are presented in Figure 3, Figure 4, Figure 5, Figure 6, Figure 7 and Figure 8.

Figure 3, Figure 4 and Figure 5 illustrate the evolution of the return (Equation (1)) over the specified time periods. Importantly, the range of the mean return values is the smallest prior to the periods of the global spread of SARS-CoV-2, as depicted in Figure 3 (mean ∈ (−0.02, 0.02)). During the the global spread of SARS-CoV-2, as seen in Figure 4, this range expanded significantly to span (−0.1, 0.1), approximately five times larger. The greatest fluctuations were observed in March 2020, marking the pandemic’s onset. In April and May, fluctuations in mean values were reduced, staying within (−0.05, 0.05). In the post-COVID period shown in Figure 5, the mean return range was doubled compared with pre-COVID values, within (−0.04, 0.04). Given that the set of analyzed time series comprised 493 items, the differences within the set were examined by standard deviation at each point. For the pre-COVID period, the standard deviation range was (0.01, 0.02), with the exception of the one point at the end of April 2018. In contrast, during the pandemic, the differences between companies’ returns were approximately four times higher, falling within the interval (0.02, 0.08), particularly in March 2020. In the post-COVID phase, standard deviation fluctuations decreased compared with that during the pandemic but were still larger than that in the pre-COVID phase, within (0.015, 0.03). Comparing the mean and standard deviation trends of the analyzed set suggests a working hypothesis that pandemic effects have modified stock market time series evolution, but as restrictions have eased, signs of recovery are emerging.

While analyzing the mean value and standard deviation of momentum evolution, certain observations appear consistent, as depicted in Figure 6, Figure 7 and Figure 8. Of particular interest are the changes in the range of fluctuations. During the pandemic, fluctuations in mean momentum were nearly quintuple those noted in the periods before and after the COVID pandemic. Nevertheless, including volume data refines the depiction of the market’s response, causing a notable modification in the evolution of the examined time series. For instance, comparing the pre-COVID mean values in Figure 3 and Figure 6 shows that the greatest return mean fluctuations occurred between late March and early April of 2018, whereas the momentum fluctuations subsided more rapidly. Similarly, the standard deviation graphs also exhibit notable discrepancies. For example, the standard deviation of the momentum evolution (Figure 6) demonstrates two clear patterns, unlike the return plot (Figure 3), which does not. Although further exploration of these differences is possible, this paper’s main aim is to probe the correlation structure, not the statistical parameter evolution of this group of companies.

Before advancing to the next stage of the analysis, it is worth summarizing the main findings. (i) The advent of the global pandemic caused a temporary disruption to the evolution of the stock market, and (ii) the return Equation (1) and momentum Equation (2) offer notably distinct perspectives on the stock market.

## 3. Distance Analysis

The second stage of the conducted study is the cross-correlation analysis, more accurately described as a distance analysis. The task of choosing a suitable distance measure is complex. Multiple analytical methodologies have been developed to explore cross-correlations [6,9,38,39,40,41,42], and the decision is far from straightforward. As highlighted in [8,9,43], the choice of a specific distance metric can influence study results. This paper uses two distance measures: the Mantegna distance [6,12,15] Equation (3) and the Manhattan distance Equation (5), also known as the taxicab distance [9,44]. The Mantegna distance is one of the widely used in the econophysics literature [6,39,45,46]. The Mantegna distance is primarily associated with portfolio analysis [47,48], which is based on statistical correlations among companies. It can be derived as the Euclidean distance between time series [21]. Therefore, its application is driven by the investor needs. However, it should be noted that the Mantegna distance is based on the Pearson linear correlation coefficient and the results close to 0 indicates a linear correlation, whereas a value equal to 1 indicates a lack of correlation, and the distance of $\sqrt{2}$ signifies an anti-linear correlation.

(3) $$d{\left(A,B\right)}_{\left(t_{1},t_{2}\right)}=\sqrt{1-\rho {\left(A,B\right)}_{\left(t_{1},t_{2}\right)}}$$

where A,B are the time series; $\left(t_{1},t_{2}\right)$ the interval; and ρ(·,·) the Pearson linear correlation coefficient.

(4) $$\rho {\left(A,B\right)}_{\left(t_{1},t_{2}\right)}=\frac{\langle AB\rangle -\langle A\rangle \langle B\rangle }{\sqrt{\langle A^{2}\rangle -{\langle A\rangle }^{2}}\sqrt{\langle B^{2}\rangle -{\langle B\rangle }^{2}}}$$

where 〈·〉 denotes the mean value of the given set.

The alternative distance is the Manhattan distance Equation (5).

(5) $$MD{\left(A,B\right)}_{\left(t_{1},t_{2}\right)}\sum_{t=t_{1}}^{t_{2}}\left|A\left(t\right)-B\left(t\right)\right|$$

In contrast to the Mantegna distance Equation (3), the Manhattan distance does not focus on the linear correlations. Instead, it allows for the observation of other dependencies [8,9,46,49].

## 4. Network Analysis

The economic system is a complex system, and as a result, network analysis is a frequently employed method, particularly in the context of cross-correlation analysis. The primary objective of network analysis is to identify the dominant relationships among the entities. The most frequently employed technique is the minimum spanning tree (MST) (see, for example, [6,17,50,51]). The primary benefit of the MST algorithm is its capacity to markedly diminish the number of links in a network, ensuring that the network is connected and that no nodes are isolated. In general, the algorithm effectively categorizes companies into their corresponding industries. The primary disadvantage of the MST is the introduction of bias due to the imposed structure. To illustrate, the MST does not encompass loops or cliques. As a consequence, the MST restricts the scope of the research. Alternatives to the MST include the principal component analysis (PCA) and the K-means algorithm [52,53,54], which can be applied to the MST or the raw distance matrix.

This paper employs an alternative methodology. Instead of building a specific network, the proposed algorithm is based on the threshold and divides the companies into three groups: (i) strongly, (ii) typically, and (iii) weakly correlated.

The networks are constructed according to the algorithm.

**Strongly** **correlated**  A connection between the companies is established if the distance between them is not greater than the first quartile of the distances on the appropriate distance matrix.
**Typically** **correlated**  The companies are connected on the network if the distance between them is greater than the first quartile and shorter than the third quartile of the distances on the appropriate distance matrix.
**Weakly** **correlated**  The companies are connected on the network if the distance between them is greater than the third quartile of the distances on the appropriate distance matrix.

In the analysis of constructed networks, the distance probability distribution is initially examined, followed by an investigation of the parameters: the degree probability distribution, the degree rank plot, the clustering coefficient distributions, and the rank clustering plot. These parameters are considered in relation to the networks’ levels of interaction, which are classified as strong, typical, or weak.

For the reader’s convenience, the aforementioned parameters are defined shortly, and their usage is justified. The degree of a node is defined as the number of edges connected to that node. The node degree distribution is a widely employed concept in network theory, as it enables the differentiation of frequently observed networks, including random networks, scale-free networks, small-world networks, and others [55,56,57,58]. This is a crucial parameter that serves to differentiate strongly connected networks. To illustrate, a road network will typically exhibit low-degree nodes (e.g., [59,60]). This is largely attributable to the high cost of constructing a new road. In contrast, an air route network will often display high-degree nodes (e.g., [61,62]), given that the development of a new airport is a costly endeavor.

In many cases, the node rank distribution is analyzed instead of the node degree distribution [63,64], particularly in the context of scale-free networks, where the power law of node ranks is anticipated. The second significant attribute of the network under examination is the clustering coefficient. It is defined by Equation (6) [65,66,67,68]. The clustering coefficient measures the density of connections between nodes, enabling the observation of structural changes in corporate networks.

(6) $$c\left(i\right)=\frac{2T(i)}{deg(i)(deg(i)-1)}$$

where T(i) is the number of triangles through node *i*, and deg(i) is the degree of node *i*.

## 5. Data Analysis

### 5.1. Statistical Properties of Distance Analysis

**Figure 9:**
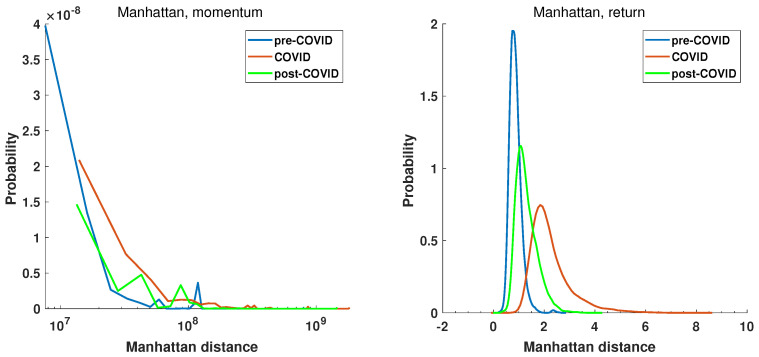
The probability distribution of Manhattan distance among the return and momentum time series of the selected companies at pre-COVID, COVID, and post-COVID intervals.

**Figure 10:**
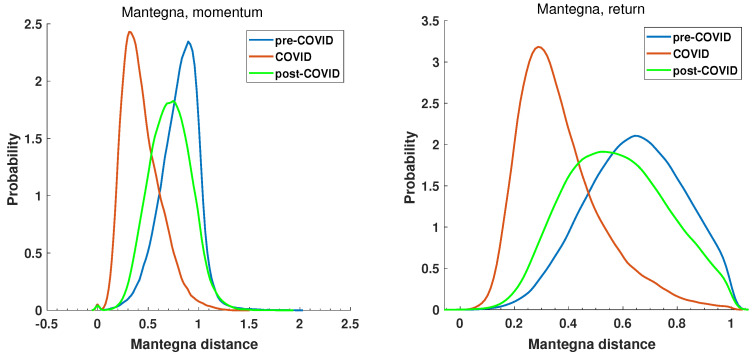
Probability distribution of Mantegna distance among the return and momentum time series of the selected companies at pre-COVID, COVID, and post-COVID intervals.

The initial step is to examine the statistical characteristics of the distance matrices. For the defined periods pre-COVID, COVID, and post-COVID, the distance matrices were calculated for the Manhattan Equation (5) and the Mantegna Equation (3) distances. The distributions’ probability density was estimated by the kernel function [69] and is presented in Figure 9 and Figure 10, which show the return and momentum time series, respectively. The most notable discrepancy between the plots in Figure 9 and Figure 10 is the magnitude of the calculated distances. The elevated values of the Manhattan distance observed in the context of the momentum time series can be attributed to the methodology employed, whereby the returns are aggregated by multiplying them by the volume. This results in the generation of relatively substantial values.

The plot of the probability distribution of Manhattan distance for the momentum time series is more difficult in interpretation. This is due to the range of distances observed. Despite the fact that the distance matrix comprises 493 companies and, therefore, 121,278 unique distances, it is insufficient to obtain a smooth distribution function for data spanning over 2 orders of magnitude. However, the common feature of all plots in Figure 9 and Figure 10 is that the COVID period probability distribution plots are different from pre- and post-COVID curves. The maximum of the Manhattan distance probability distribution function (PDF) of the return time series of the COVID period in Figure 9 is located at a greater distance than that of the pre- and post-COVID time series where the value of the maximum of PDF is smaller. The COVID-19 pandemic caused a shift towards greater distances and an increase in variance. The post-COVID Manhattan distance PDF curve (green line) appears to be reverting to the pre-COVID state (blue line). It is noteworthy that the Manhattan distance values observed in the pre-COVID periods are concentrated around zero, which is characteristic of a stable market situation. This evidence lends support to the selection of this period as a reference point. The second reference period, namely, the post-COVID period, also exhibits a maximum close to zero, indicating that the market state is likely to revert to a normal evolution. In this state, companies’ stocks evolve in a similar manner, and the difference in return time series is low, as shown in Equation (5).

Furthermore, the recovery of the distance to the pre-COVID state is also evident in Figure 10, which depicts the Mantegna distance. However, the information obtained by Mantegna distance (Equation (3)) is substantially different, as it is based on the Pearson correlation coefficient, which indicates whether the relationship between the time series can be explained by a linear function. The probability density functions of the considered intervals in the case of the momentum and return time series are presented in Figure 10.

The pre-COVID, COVID, and post-COVID periods are represented by blue, read, and green curves, respectively. It is noteworthy that the PDF curve for the COVID period, for both the momentum and the return time series, is distinctly separated from the reference periods. The maximum of the momentum time series PDF is d≈0.3, while that of pre-COVID is d≈0.9 and that of post-COVID d≈0.7. A similar observation can be made for the return time series. The maximum of the Mantegna distance PDF decreases to a value of d≈0.25, while in pre-COVID it is d≈0.65 and in post-COVID d≈0.5. A second noteworthy observation is the distinction in the insights yielded by the analysis of the momentum and return time series, as illustrated in Figure 10. In the momentum time series PDF plot, the maximum of the pre-COVID curve is observed at d≈0.9 and reaches d≈1.4, indicating that the time series are not correlated or even anti-correlated. In contrast, during the pandemic (red curve), the maximum is shifted towards d≈0, which means highly correlated time series. A similar conclusion cannot be drawn from the return time series analysis, where the pre-COVID and post-COVID curves display significantly higher dispersion and no anti-correlations, resulting in distances greater than 1. The momentum graph seems to be capturing the flow of capital on stock markets. Of particular interest is the curve representing the period of the pandemic, which demonstrates a high degree of correlation among the time series. This is the consequence of a markedly robust external influence, which is affecting the market and causing the shares to behave in a congruent manner.

### 5.2. Network Properties—Degree Distribution

The distance matrices obtained for the Manhattan Equation (5) and the Mantegna Equation (3) distances are analyzed by constructing threshold-based networks, which are used to identify the companies that are correlated strongly, typically, and weakly. As each network comprises 493 nodes, the graph in traditional format in this paper would be illegible. Therefore, the plots are provided as Supplementary Files in the vector graphics format.

**Figure 11:**
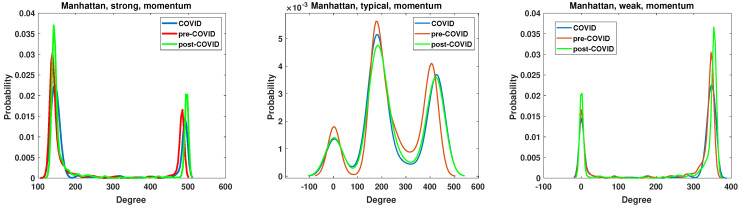
The node degree PDF for the Manhattan distance between the momentum time series for the strongly, typically, and weakly correlated companies.

**Figure 12:**
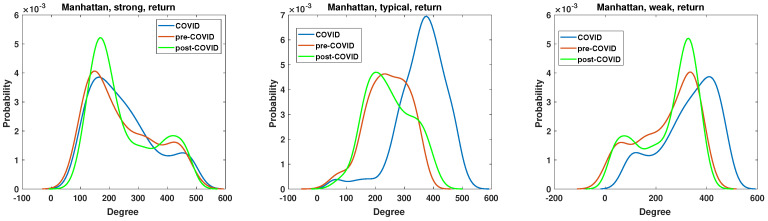
The node degree PDF for the Manhattan distance between the return time series for the strongly, typically, and weakly correlated companies.

**Figure 13:**
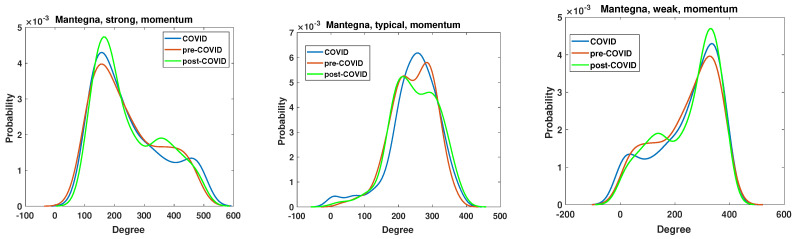
The node degree PDF for the Mantegna distance between the momentum time series for the strongly, typically, and weakly correlated companies.

**Figure 14:**
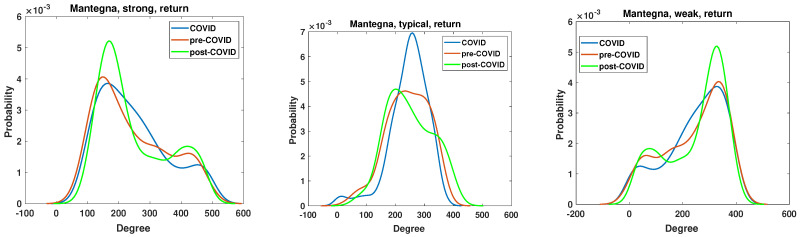
The node degree PDF for the Mantegna distance between the return time series for the strongly, typically and weakly correlated companies.

The node degree distribution properties of the generated networks are analyzed by the kernel function PDF estimation. The results are presented in Figure 11, Figure 12, Figure 13 and Figure 14. A chi-square test was used to determine whether the apparent differences in the node degree distributions were statistically significant. In all cases (Manhattan and Mantegna distances as well as for all considered networks and intervals), the hypothesis of no difference among distributions was rejected (at the significance level *p* = 0). The structure of the networks based on the Manhattan distance and momentum time series is illustrated in Figure 11. The plot demonstrates that the proposed cut-off points result in substantially different structures. The degree distribution of strongly correlated companies exhibits two distinctive maxima, indicating the existence of two distinct groups among strongly correlated companies. The first group comprises nodes with 150 links, while the second group is characterized by nodes that are connected to all other nodes. It is notable that during the period of the pandemic, the maximum values decreased, whereas in the subsequent period, the local maximum values reached their highest levels. The companies that are typically correlated exhibit three distinctive maxima, along with a group of disconnected nodes. The second group of companies is characterized by ≈180 edges, while the last distinguished group comprises nodes with ≈400 edges. In the network of typically correlated companies, the pre-COVID curve differs from the post-COVID and pre-COVID PDFs. In the pre-COVID period, the probability of observing disconnected nodes is approximately 0.002, while in the post-COVID and pre-COVID periods, this probability is approximately 0.0012, indicating a lower probability of observing disconnected nodes. Similarly, the highly connected nodes exhibit almost identical COVID and post-COVID PDF curves, while the pre-COVID maximum is greater. The curves for the group of disconnected nodes and the nodes with the highest number of edges are almost identical for the period of the pandemic and the subsequent period of recovery. They differ from the pre-pandemic network. Therefore, it can be concluded that the system did not revert to its pre-COVID state.

The network of the weakly correlated companies, based on the momentum time series analyzed by the Manhattan distance, exhibits a degree probability density function (PDF) plot that is similar to that of a strongly connected network, displaying two distinctive peaks. However, the values at which the peaks are observed differ. The first group comprises disconnected nodes, while the second group consists of nodes with 360 edges, which are not connected to all the remaining nodes, as observed in the strongly correlated network.

The node degree PDF of the network constructed based on the Manhattan distance among the return time series is presented in Figure 12. The structure of the networks constructed based on the Manhattan distance of the return time series differs significantly from that based on the momentum time series. The PDF of the node degree of the strong and weak networks does not exhibit a two-state system but a whole range of values. The second substantial difference is that, in the case of the typically correlated time series network in Figure 12, the COVID curve is disparate from pre- and post-COVID PDFs. The pre-COVID node degree PDF of the typically correlated network has a single broad maximum beginning at the degree ≈200 and ends at the degree ≈300. For the COVID period, the degree PDF is more concentrated, presenting a typical maximum at the degree ≈380. Therefore, the maximum is shifted to higher values, and a small group of nodes with a degree in the range between 50 and 200 links (a flat part of the blue curve) is also present. In the case of the period of the pandemic, the degree probability density function is more concentrated, exhibiting a typical maximum at a degree of approximately 380. Therefore, the maximum is shifted to higher values, and a small group of nodes with a degree between 50 and 200 (a flat part of the blue curve) is also present. It can be concluded that, with regard to the return time series, the greatest changes in network structure are observed among the typically correlated companies. The node degree PDF of strong and weak networks exhibits the most significant discrepancies in the post-COVID period. In the case of the robust network, the initial peak of the node degree probability distribution is observed at a degree value of 190, with a probability of p=0.0052, which is significantly higher than in other periods, where p≈0.004. Therefore, following the pandemic, there has been an increase in the number of companies with relatively few edges. In contrast, the situation for the weak network is characterized by a “complementary” dynamic. Furthermore, an increase in the number of nodes with a high degree is evident. Additionally, the maximum of the PDF is observed to be beyond 400 edges.

The Mantegna distance-based analyses of the node degree distribution are presented in Figure 13 and Figure 14, which depict the momentum and return time series, respectively. The networks were generated based on the value of the Pearson correlation coefficient, which represents the level of linearity among the time series. The greatest alterations in node degree probability density function (PDF) resulting from the advent of the SARS-CoV-2 pandemic are observed in the case of both weak and typical networks. The node degree PDF of the strong network demonstrates relatively small changes resulting from the global impact of the SARS-CoV-2 pandemic. The observed changes are concentrated at two specific values. The initial peak is observed for the degree ≈160, where the pre-COVID maximum has a value of p=0.004, for the COVID curve p=0.0042, and for the post-COVID curve p=0.0048. Therefore, the probability of observing nodes with a relatively low number of edges increased during the period of the pandemic and did not revert to the previous state. This observation differs from that presented in the Manhattan picture in Figure 11, where the peak associated with the pandemic is the lowest and the system appears to revert to a state that existed prior to the advent of the pandemic. The typical network structure exhibits a distinct behavior pattern in comparison with the strong network, as illustrated in Figure 13. The primary distinction between the pre- and post-COVID network is that the former exhibits two modes, whereas the latter displays a single maximum in the node degree distribution. Furthermore, at the initial peak (node degree = 210), the system reverted to its previous state. Conversely, at the second maximum (node degree = 290), the probability of observing a node decreased from 0.0058 in the pre-COVID period to 0.0048. The weak network, comprising companies with the highest distance and lowest correlations, exhibits a mirror reflection of the strong network case. In this case, the most frequently observed node degree is 320 edges, and the pre-COVID time series exhibit a lower value of the maximum point. In contrast, the maximum increase occurs during the pandemic, while the post-COVID line displays the greatest value of the maximum. Consequently, the number of nodes with high edges attached increased during and after the pandemic. This finding aligns with the output of the strongly correlated network, where the number of nodes with a relatively low number of edges increased. Therefore, it can be concluded that the measures taken during the pandemic decreased the correlations in the Mantegna picture applied to the momentum time series.

The return time series analyzed by Mantegna distance are frequently employed in the field of econophysics, as evidenced by numerous studies e.g., [4,9,16]. The node degree PDF is presented in Figure 14. The most pronounced impact of the SARS-CoV-2 pandemic is observed in the typical network, where the node degree PDF is markedly elevated in comparison with the pre-COVID and post-COVID periods. In contrast, the strong and weak networks exhibit markedly different patterns, with the greatest shifts occurring in the post-COVID period. In the case of the strong and weak networks, in the post-COVID period, the maximum value of the node degree PDF is observed at p=0.0052, whereas in the pre-COVID and COVID periods, this value does not exceed p=0.004. This indicates that the effects of the pandemic have resulted in alterations to the structural characteristics of the strong and weak networks.

The principal insight derived from the structural analysis based on the node degree distribution is that the proposed modifications, namely, the analysis of the momentum time series, the introduction of the Manhattan distance, and utilization of a network based on the threshold, provide a novel perspective on the system analysis. The return-based analysis indicates that the pandemic has resulted in significant and enduring alterations to the network structure, in terms of both its strong and weak components. In contrast, the momentum time series-based analysis suggests that, following the initial disturbance, the system has reverted to its previous state. It is evident that these findings require a comprehensive economic analysis of the market, which is beyond the scope of this paper. Nevertheless, it seems reasonable to posit that, in the situation subsequent to the lifting of restrictions, the previous structure should be rebuilt. Therefore, the results of the momentum time series-based analysis appear to be more reliable.

### 5.3. Network Properties—Clustering Coefficient

**Figure 15:**
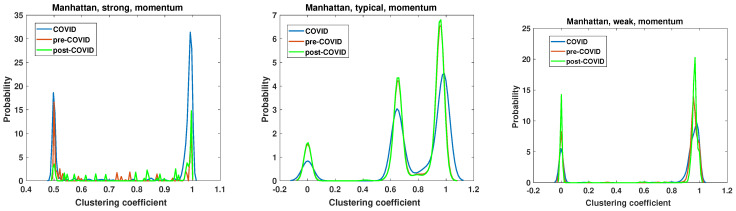
Clustering coefficient probability distribution of the strong, typical, and weak network obtained for the Manhattan distance matrix of the momentum time series.

**Figure 16:**
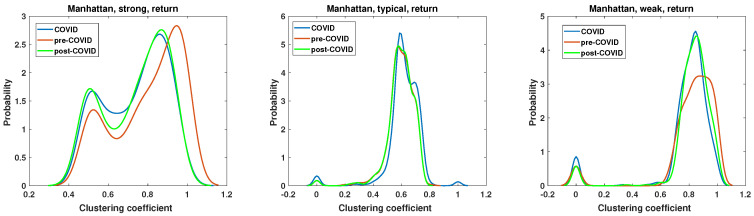
Clustering coefficient probability distribution of the strong, typical, and weak network obtained for the Manhattan distance matrix of the return time series.

**Figure 17:**
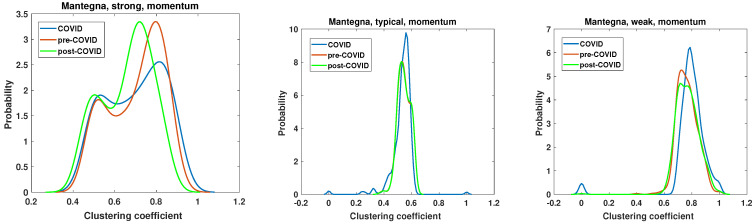
Clustering coefficient probability distribution of the strong, typical, and weak network obtained for the Mantegna distance matrix of the momentum time series.

**Figure 18:**
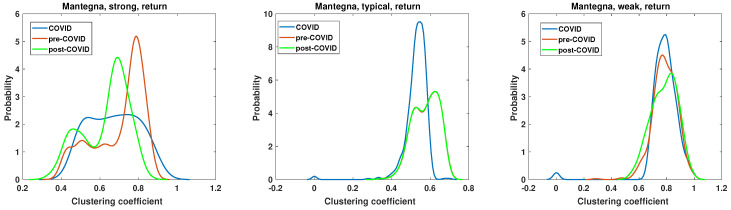
Clustering coefficient probability distribution of the strong, typical, and weak network obtained for the Mantegna distance matrix of the return time series.

The second parameter under examination is the clustering coefficient. This parameter characterizes the density of connections and is equal to 1 for a fully connected clique. This allows for the verification of whether the network is composed of nodes that are closely related. The results are presented in Figure 15, Figure 16, Figure 17 and Figure 18. The clustering coefficient was calculated according to Equation (6). It is crucial to emphasize that, in accordance with the established definition, the clustering coefficient is calculated for each individual node within the network. Given the considerable size of the network, it was feasible to estimate the probability density of the clustering coefficient. Similarly, as with the analysis of node degree, the kernel function [69] was employed.

The clustering coefficient analysis results of strong, typical, and weak networks obtained for the Manhattan distance matrices based on the momentum time series are presented in Figure 15. They support the findings of the node degree analysis presented in Figure 11. The chi-square test applied to the clustering coefficient distributions for the momentum time series analyzed by Manhattan distance shows that the hypotheses that there are no differences among the distributions must be rejected at the significance level p≈0. In both the strong and weak networks, two distinct groups emerge as the dominant ones. In the case of the strong network, two distinct types of nodes emerge as particularly prevalent during the periods of the pandemic. The first group is characterized by a clustering coefficient of c=0.5, while the second is characterized by a clustering coefficient of c=1, which denotes a clique of nodes. During the period of the pandemic, the number of fully connected nodes increased markedly. It is noteworthy that the nodes with c=0.5 in the pre-COVID and post-COVID periods have the same value, whereas in the pre-COVID period, the number of nodes with c=0.5 is significantly smaller. Despite apparent similarities, the weak network analysis differs from the strong network clustering coefficient PDF. Once more, two principal groups emerge, although the initial set of nodes exhibits a clustering coefficient of zero, indicating a lack of connectivity. In the post-COVID period, the number of such nodes rises. A similar effect is observed for the second type of nodes with c≈1, which can be considered members of cliques. Furthermore, the maximum value of the clustering coefficient PDF was observed to have increased in the post-COVID period. The structure and evolution of the clustering coefficient of the typical network based on the Manhattan distance of the momentum time series differ from those observed in the weak and strong networks. The network can be divided into three classes of nodes based on their clustering coefficient: the disconnected nodes (with c≈0), the nodes on a relatively dense network (c≈0.65), and the cliques (with c≈1). It is noteworthy that this division is observed to be stable across all considered intervals. Only the magnitude of PDF is changed, and in the COVID period, the maximum values are significantly lower. It can be noted that the pre-COVID and post-COVID PDFs are almost identical, indicating that the structure of the typical network is recovering from its previous state after the COVID-19 pandemic. This observation differs from that of strong and weak networks, where the system in the post-COVID period differs from that of the pre-COVID interval.

The results of the clustering coefficient analysis of the strong, typical, and weak networks, obtained on the Manhattan distance matrix based on the return time series, are presented in Figure 16. The sole distinction between the momentum and return time series lies in the volume, which significantly influences the alterations in the clustering coefficient structure. In the case of a typical network, the changes in the clustering coefficient PDF are negligible, which is a surprising outcome that is contradictory to the momentum time series case in Figure 15. The chi-square test results are presented in Table 1. They show that, except for the pre-COVID vs. COVID and pre-COVID vs. post-COVID, in the case of the clustering coefficient distributions of strongly correlated companies’ network, there are no pairs of distributions whose differences are statistically justified.

The two remaining network structures, namely, the strong and weak networks, exhibit some changes resulting from the pandemic. Notably, there has been a modification to the pre-COVID clustering coefficient PDF. In the case of the strong network, the maximum of PDF at COVID and post-COVID is shifted towards lower values c≈0.9, so the density of connections slightly decreases. Additionally, the second peak exhibits slightly elevated values, increasing from p=1.4 to p=1.6. Thus, the probability of encountering nodes with a clustering coefficient of c=0.5 increases for the COVID and post-COVID periods. The clustering coefficient PDF for the weak network in Figure 16 has two distinct maxima of different highs. The first group of nodes for c=0, so the disconnected nodes during pre-COVID and post-COVID reach a value of p=0.5, while for COVID, p=0.9. This indicates that the number of disconnected nodes increased during the pandemic. A similar observation can be made for the typical network at the group of disjoint nodes. The second maximum of the weak network clustering coefficient PDF is observed at c=0.8. However, its value for the COVID and post-COVID periods is p=4.5, while in the pre-COVID period, it is p=3.1. Therefore, the probability of observing highly connected nodes is increased by the pandemic, a feature that persists in the post-pandemic period.

The results of the momentum time series analysis based on the Mantegna distance are presented in Figure 17. In this case, only the differences between pre-COVID and COVID clustering coefficient distribution for the weak network are statistically significant. However, the key differences observed in this type of analysis are worth discussing. The chi-square test results of the clustering coefficient distributions for networks based on the momentum time series analyzed by the Mantegna distance are presented in Table 2. The PDF of the clustering coefficient for the strong graph based on the Mantegna distance among the momentum time series shows clear changes caused by the COVID-19 pandemic. The pre- and post-COVID PDFs are quite similar in shape, but the maximum of those functions is separated by c=0.1. Consequently, in the post-COVID period, the most probable node type has a lower clustering coefficient than in the pre-COVID time. The COVID period in this plot (i.e., Mantegna, strong, momentum) differs substantially from those of the reference intervals. In particular, the maximum of the clustering coefficient distribution is lower at p≈2.6 compared with the pre- and post-COVID periods, where it is p≈3.4. In the case of the typical graph, the pre- and post-COVID PDFs of the clustering coefficient are identical. Therefore, for this specific type of network and distance measure, the system’s structure reverted to its initial state. The only discrepancy observed in the typical network is in the plot of the pandemic period, where the maximum is markedly higher, reaching p≈9.8, while for the other two curves, p=8. The weak network, which focuses on the longest distances among Mantegna distances for momentum time series, does not demonstrate significant effects of the pandemic. The maximum of the clustering coefficient is increased by Δc=0.1 for the COVID period, in comparison with that for the pre- and post-COVID periods. Furthermore, the maxima for pre- and post-COVID are roughly the same, but the magnitude decreased in the post-COVID interval by Δp=0.56. A second notable aspect of the weak graph is that the set of c=0 is non-negligible during the COVID interval.

The results of the clustering coefficient analysis for the most typical combination of methods, namely, Mantegna distance applied to the return time series, are presented in Figure 18. In this case, only the pre-COVID vs. COVID clustering coefficient distributions are statistically different according to the results of the chi-square test, as shown in Table 3. However, some features and differences are particularly noticeable. The clustering coefficient of the strong graph in the pre-COVID period is presented by the red line, which exhibits a dominant maximum at c=0.8 with a value of p=5.1. During the COVID period, the clustering coefficient PDF changes, strongly broadening the range of observed values. In this instance, the clustering coefficient PDF is characterized by a markedly flat and broad range, c∈(0.5,0.8) with a value of p≈2.2. Therefore, during the pandemic, no dominant class of nodes was observed. Following the pandemic, the system exhibited a notable recovery in many pre-COVID features. The clustering coefficient PDF demonstrated a distinct maximum at c=0.7, which exhibited a slight shift from the pre-COVID maximum and was slightly lower. Additionally, a second maximum was observed at c=0.45, suggesting the existence of two distinct classes of nodes with different densities of connections. The second type of network, the typical graph, exhibits similarities to analogous networks that have been previously analyzed. The primary observation is that the clustering coefficient PDF of the pre- and post-COVID periods are identical, indicating that the structure of the graph in terms of edge density is consistent. The effects of the pandemic are evident in these significant alterations. The maximum of the period during the pandemic is located at c=0.55, with the height of this peak being p=9.4. In contrast, in the pre- and post-pandemic periods, the maximum was p=5.4. The circumstances of the pandemic resulted in the unification of nodes with a clustering coefficient that exhibited a single dominant value with minimal dispersion. This observation contrasts with that of a strong network, where the pandemic facilitated the dissemination of the distribution and the proliferation of a broad range of nodes observed on the network. The final network analyzed was that of a weak network, as shown in Figure 18. In this case, there was in fact a single type of node with a clustering coefficient within the interval c∈(0.6,1). The visual difference among the considered intervals is the high of the maximum. For pre-COVID, p=4.5; for COVID, p=5.2; and for post-COVID, p=3.8.

## 6. Conclusions

The principal objective of this study was to investigate the cross-correlations among companies representative of the US economy and the influence of the coronavirus pandemic on the structure of interdependencies. This study was conducted on a sample of companies listed in the S&P 500 Index. It employed cross-correlation analysis utilizing established techniques, including return series and Mantegna distance, in addition to a novel approach based on Manhattan distance and momentum series. This method considers not only returns but also stock market trading volume. The primary objective of the modifications was to mitigate the constraints imposed by the conventional approach, namely, to prioritize linear correlations and exclude the consideration of market reactions as reflected in stock turnover levels. The structural alterations were examined through the construction of three categories of networks based on a specified threshold: networks of strongly, typically, and weakly correlated entities.

The analysis showed that the global COVID-19 pandemic (and the measures taken by the authorities) caused substantial alterations to the analyzed network’s structure. As expected, the changes were temporary, and the structures of the investigated networks before and after the COVID-19 pandemic were similar. It can be concluded that the system has essentially returned to its pre-pandemic state and the implemented measures have ensured the preservation of the market’s fundamental structure. Additionally, it can be confirmed that the hypothesis regarding the necessity of incorporating trading volumes into the analytical process has been validated. The observed networks demonstrated notable discrepancies in their structural characteristics when examined across momentum and return time series as well as distances. Despite the consensus that the post-pandemic market has demonstrated recovery from the pandemic, the inclusion of trading volumes is essential for a comprehensive understanding of the market’s structural and macroeconomic attributes.

## Figures and Tables

**Table 1:** The statistical significance for rejecting the null hypothesis that there is no difference between clustering coefficient distributions for the networks based on the Manhattan distance of the return time series. Cases with *p* > 0.01 are marked in bold font.

	Weak	Typical	Strong
Pre-COVID vs. COVID	$3.27\times 10^{-143}$	$3.17\times 10^{-12}$	0.0815
COVID vs. Post-COVID	$1.13\times 10^{-101}$	0.0029	$1.9\times 10^{-8}$
Pre-COVID vs. Post-COVID	$2.24\times 10^{-133}$	0.0032	0.2219

**Table 2:** The statistical significance for rejecting the null hypothesis that there is no difference between clustering coefficient distributions for the networks based on the Mantegna distance of momentum time series. Cases with *p* > 0.01 are marked in bold font.

	Weak	Typical	Strong
Pre-COVID vs. COVID	$1.85\times 10^{-10}$	0.0184	0.2731
COVID vs. Post-COVID	0.0205	0.2416	0.2425
Pre-COVID vs. Post-COVID	0.0872	0.2423	0.2423

**Table 3:** The statistical significance for rejecting the null hypothesis that there is no difference between clustering coefficient distributions for the networks based on the Mantegna distance of the return time series. Cases with *p* > 0.01 are marked in bold font.

	Weak	Typical	Strong
Pre-COVID vs. COVID	$1.49\times 10^{-10}$	0.0176	0.2586
COVID vs. Post-COVID	0.2048	0.2412	0.2459
Pre-COVID vs. Post-COVID	0.2549	0.2423	0.2480

## Data Availability

The data used in this study are available at the Yahoo Finance web page https://finance.yahoo.com (accessed on 10 June 2024).

## Supplementary Materials

The following supporting information can be downloaded at https://www.mdpi.com/article/10.3390/e27020148/s1. Figure S1: Network of strongly correlated companies, Manhattan distance, momentum time series, pre-COVID period; Figure S2: Network of strongly correlated companies, Mantegna distance, momentum time series, pre-COVID period; Figure S3: Network of strongly correlated companies, Manhattan distance, return time series, pre-COVID period; Figure S4: Network of strongly correlated companies, Mantegna distance, return time series, pre-COVID period; Figure S5: Network of strongly correlated companies, Manhattan distance, momentum time series, COVID period; Figure S6: Network of strongly correlated companies, Mantegna distance, momentum time series, COVID period; Figure S7: Network of strongly correlated companies, Manhattan distance, return time series, COVID period; Figure S8: Network of strongly correlated companies, Mantegna distance, return time series, COVID period; Figure S9: Network of strongly correlated companies, Manhattan distance, momentum time series, post-COVID period; Figure S10: Network of strongly correlated companies, Mantegna distance, momentum time series, post-COVID period; Figure S11: Network of strongly correlated companies, Manhattan distance, return time series, post-COVID period; Figure S12: Network of strongly correlated companies, Mantegna distance, return time series, post-COVID period; Figure S13: Network of typically correlated companies, Manhattan distance, momentum time series, pre-COVID period; Figure S14: Network of typically correlated companies, Mantegna distance, momentum time series, pre-COVID period; Figure S15: Network of typically correlated companies, Manhattan distance, return time series, pre-COVID period; Figure S16: Network of typically correlated companies, Mantegna distance, return time series, pre-COVID period; Figure S17: Network of typically correlated companies, Manhattan distance, momentum time series, COVID period; Figure S18: Network of typically correlated companies, Mantegna distance, momentum time series, COVID period; Figure S19: Network of typically correlated companies, Manhattan distance, return time series, COVID period; Figure S20: Network of typically correlated companies, Mantegna distance, return time series, COVID period; Figure S21: Network of typically correlated companies, Manhattan distance, momentum time series, post-COVID period; Figure S22: Network of typically correlated companies, Mantegna distance, momentum time series, post-COVID period; Figure S23: Network of typically correlated companies, Manhattan distance, return time series, post-COVID period; Figure S24: Network of typically correlated companies, Mantegna distance, return time series, post-COVID period; Figure S25: Network of weakly correlated companies, Manhattan distance, momentum time series, pre-COVID period; Figure S26: Network of weakly correlated companies, Mantegna distance, momentum time series, pre-COVID period; Figure S27: Network of weakly correlated companies, Manhattan distance, return time series, pre-COVID period; Figure S28: Network of weakly correlated companies, Mantegna distance, return time series, pre-COVID period; Figure S29: Network of weakly correlated companies, Manhattan distance, momentum time series, COVID period; Figure S30: Network of weakly correlated companies, Mantegna distance, momentum time series, COVID period; Figure S31: Network of weakly correlated companies, Manhattan distance, return time series, COVID period; Figure S32: Network of weakly correlated companies, Mantegna distance, return time series, COVID period; Figure S33: Network of weakly correlated companies, Manhattan distance, momentum time series, post-COVID period; Figure S34: Network of weakly correlated companies, Mantegna distance, momentum time series, post-COVID period; Figure S35: Network of weakly correlated companies, Manhattan distance, return time series, post-COVID period; Figure S36: Network of weakly correlated companies, Mantegna distance, return time series, post-COVID period.

## Abbreviations

The following abbreviations are used in this manuscript:

PDF	probability distribution function
MST	minimum spanning tree
MD	Manhattan distance
d	Mantegna distance

## Appendix A

The list of companies considered in the analysis includes A, AAL, AAPL, ABBV, ABT, ACGL, ACN, ADBE, ADI, ADM, ADP, ADSK, AEE, AEP, AES, AFL, AIG, AIZ, AJG, AKAM, ALB, ALGN, ALL, ALLE, AMAT, AMCR, AMD, AME, AMGN, AMP, AMT, AMZN, ANET, ANSS, AON, AOS, APA, APD, APH, APTV, ARE, ATO, AVB, AVGO, AVY, AWK, AXON, AXP, AZO, BA, BAC, BALL, BAX, BBWI, BBY, BDX, BEN, BF-B, BG, BIIB, BIO, BK, BKNG, BKR, BLDR, BLK, BMY, BR, BRK-B, BRO, BSX, BWA, BX, BXP, C, CAG, CAH, CAT, CB, CBOE, CBRE, CCI, CCL, CDNS, CDW, CE, CF, CFG, CHD, CHRW, CHTR, CI, CINF, CL, CLX, CMCSA, CME, CMG, CMI, CMS, CNC, CNP, COF, COO, COP, COR, COST, CPAY, CPB, CPRT, CPT, CRL, CRM, CRWD, CSCO, CSGP, CSX, CTAS, CTLT, CTRA, CTSH, CTVA, CVS, CVX, CZR, D, DAL, DAY, DD, DE, DECK, DFS, DG, DGX, DHI, DHR, DIS, DLR, DLTR, DOC, DOV, DOW, DPZ, DRI, DTE, DUK, DVA, DVN, DXCM, EA, EBAY, ECL, ED, EFX, EG, EIX, EL, ELV, EMN, EMR, ENPH, EOG, EPAM, EQIX, EQR, EQT, ES, ESS, ETN, ETR, ETSY, EVRG, EW, EXC, EXPD, EXPE, EXR, F, FANG, FAST, FCX, FDS, FDX, FE, FFIV, FI, FICO, FIS, FITB, FMC, FOX, FOXA, FRT, FSLR, FTNT, FTV, GD, GDDY, GE, GEN, GILD, GIS, GL, GLW, GM, GNRC, GOOG, GOOGL, GPC, GPN, GRMN, GS, GWW, HAL, HAS, HBAN, HCA, HD, HES, HIG, HII, HLT, HOLX, HON, HPE, HPQ, HRL, HSIC, HST, HSY, HUBB, HUM, HWM, IBM, ICE, IDXX, IEX, IFF, INCY, INTC, INTU, INVH, IP, IPG, IQV, IR, IRM, ISRG, IT, ITW, IVZ, J, JBHT, JBL, JCI, JKHY, JNJ, JNPR, JPM, K, KDP, KEY, KEYS, KHC, KIM, KKR, KLAC, KMB, KMI, KMX, KO, KR, L, LDOS, LEN, LH, LHX, LIN, LKQ, LLY, LMT, LNT, LOW, LRCX, LULU, LUV, LVS, LW, LYB, LYV, MA, MAA, MAR, MAS, MCD, MCHP, MCK, MCO, MDLZ, MDT, MET, META, MGM, MHK, MKC, MKTX, MLM, MMC, MMM, MNST, MO, MOH, MOS, MPC, MPWR, MRK, MRNA, MRO, MS, MSCI, MSFT, MSI, MTB, MTCH, MTD, MU, NCLH, NDAQ, NDSN, NEE, NEM, NFLX, NI, NKE, NOC, NOW, NRG, NSC, NTAP, NTRS, NUE, NVDA, NVR, NWS, NWSA, NXPI, O, ODFL, OKE, OMC, ON, ORCL, ORLY, OXY, PANW, PARA, PAYC, PAYX, PCAR, PCG, PEG, PEP, PFE, PFG, PG, PGR, PH, PHM, PKG, PLD, PM, PNC, PNR, PNW, PODD, POOL, PPG, PPL, PRU, PSA, PSX, PTC, PWR, PYPL, QCOM, QRVO, RCL, REG, REGN, RF, RJF, RL, RMD, ROK, ROL, ROP, ROST, RSG, RTX, RVTY, SBAC, SBUX, SCHW, SHW, SJM, SLB, SMCI, SNA, SNPS, SO, SPG, SPGI, SRE, STE, STLD, STT, STX, STZ, SWK, SWKS, SYF, SYK, SYY, T, TAP, TDG, TDY, TECH, TEL, TER, TFC, TFX, TGT, TJX, TMO, TMUS, TPR, TRGP, TRMB, TROW, TRV, TSCO, TSLA, TSN, TT, TTWO, TXN, TXT, TYL, UAL, UBER, UDR, UHS, ULTA, UNH, UNP, UPS, URI, USB, V, VICI, VLO, VMC, VRSK, VRSN, VRTX, VST, VTR, VTRS, VZ, WAB, WAT, WBA, WBD, WDC, WEC, WELL, WFC, WM, WMB, WMT, WRB, WST, WTW, WY, WYNN, XEL, XOM, XYL, YUM, ZBH, ZBRA, and ZTS.
